# Dogs Have the Most Neurons, Though Not the Largest Brain: Trade-Off between Body Mass and Number of Neurons in the Cerebral Cortex of Large Carnivoran Species

**DOI:** 10.3389/fnana.2017.00118

**Published:** 2017-12-12

**Authors:** Débora Jardim-Messeder, Kelly Lambert, Stephen Noctor, Fernanda M. Pestana, Maria E. de Castro Leal, Mads F. Bertelsen, Abdulaziz N. Alagaili, Osama B. Mohammad, Paul R. Manger, Suzana Herculano-Houzel

**Affiliations:** ^1^Instituto de Ciências Biomédicas, Universidade Federal do Rio de Janeiro, Rio de Janeiro, Brazil; ^2^Department of Psychology, University of Richmond, Richmond, VA, United States; ^3^Department of Psychiatry and Behavioral Sciences, School of Medicine, University of California, Davis, Davis, CA, United States; ^4^Biosystematics Section, Zoological Museum SNM, Copenhagen, Denmark; ^5^Centre for Zoo and Wild Animal Health, Copenhagen Zoo, Frederiksberg, Denmark; ^6^KSU Mammals Research Chair, Department of Zoology, College of Science, King Saud University, Riyadh, Saudi Arabia; ^7^School of Anatomical Sciences, Faculty of Health Sciences, University of the Witwatersrand, Johannesburg, South Africa; ^8^Department of Psychology, Vanderbilt University, Nashville, TN, United States; ^9^Department of Biological Sciences, Vanderbilt University, Nashville, TN, United States; ^10^Vanderbilt Brain Institute, Vanderbilt University, Nashville, TN, United States

**Keywords:** number of neurons, brain size, carnivorans, evolution, metabolic cost, domestication, predator-prey

## Abstract

Carnivorans are a diverse group of mammals that includes carnivorous, omnivorous and herbivorous, domesticated and wild species, with a large range of brain sizes. Carnivory is one of several factors expected to be cognitively demanding for carnivorans due to a requirement to outsmart larger prey. On the other hand, large carnivoran species have high hunting costs and unreliable feeding patterns, which, given the high metabolic cost of brain neurons, might put them at risk of metabolic constraints regarding how many brain neurons they can afford, especially in the cerebral cortex. For a given cortical size, do carnivoran species have more cortical neurons than the herbivorous species they prey upon? We find they do not; carnivorans (cat, mongoose, dog, hyena, lion) share with non-primates, including artiodactyls (the typical prey of large carnivorans), roughly the same relationship between cortical mass and number of neurons, which suggests that carnivorans are subject to the same evolutionary scaling rules as other non-primate clades. However, there are a few important exceptions. Carnivorans stand out in that the usual relationship between larger body, larger cortical mass and larger number of cortical neurons only applies to small and medium-sized species, and not beyond dogs: we find that the golden retriever dog has more cortical neurons than the striped hyena, African lion and even brown bear, even though the latter species have up to three times larger cortices than dogs. Remarkably, the brown bear cerebral cortex, the largest examined, only has as many neurons as the ten times smaller cat cerebral cortex, although it does have the expected ten times as many non-neuronal cells in the cerebral cortex compared to the cat. We also find that raccoons have dog-like numbers of neurons in their cat-sized brain, which makes them comparable to primates in neuronal density. Comparison of domestic and wild species suggests that the neuronal composition of carnivoran brains is not affected by domestication. Instead, large carnivorans appear to be particularly vulnerable to metabolic constraints that impose a trade-off between body size and number of cortical neurons.

## Introduction

*Carnivora* is a remarkable order for comparative studies of neuroanatomy because of the wide range of brain and body size of its members, from the smallest, mouse-sized least weasel to the 5-ton Southern elephant seal, overlapping with most other mammalian clades. Carnivoran brains are highly convoluted, although less so than cetartiodactyl and primate brains of similar mass (Pillay and Manger, 2007; Manger et al., 2010). Carnivorans are also highly diverse: they can be social or solitary animals; carnivorous, omnivorous, or even frugivorous; domestic (such as cats and dogs) or wild.

Carnivory comes with costs and benefits that are likely to impose a delicate balance on the relationship between brain and body. Although meat eating (and therefore hunting) is not universal among carnivorans, hunting is a feature of this clade that might impose a larger cognitive demand on the brain than its counterpart: being preyed upon, since prey species tend to find safety in numbers. It is thus tempting to predict that cognitive demand has imposed positive pressure on carnivorans for larger numbers of neurons compared to their prey species, mostly artiodactyls, of similar or even larger body and brain size. However, this possible advantage conferred by larger numbers of neurons, particularly in the cerebral cortex, would have to be balanced by the metabolic cost of having more neurons. Daily sleep requirement and dietary content are key elements here. While artiodactyls afford large bodies through a large time investment in feeding on plant leaves of low caloric content (Du Toit and Yetman, 2005), an investment made possible by brains that can do with as few as 3 h of sleep per day (Zepelin and Rechtschaffen, 1974), carnivorans typically are inactive, possibly asleep, over 12 h per day (Zepelin and Rechtschaffen, 1974). Moreover, carnivorous species have highly variable feeding success (Gorman et al., 1998), and hunting comes at a particularly high metabolic cost for the largest predator species (Carbone et al., 2007), factors which are likely to be a liability for a tissue such as brain that has a consistently high metabolic requirement, and is one of the most expensive tissues of the body (Aschoff et al., 1971). Considering that the cerebral cortex is the most expensive structure within the brain (Karbowski, 2007), and that the energetic cost of the brain is proportional to its number of neurons (Herculano-Houzel, 2011), it is conceivable that large meat-eating carnivorans are particularly subject to energetic constraints that might limit their numbers of brain neurons, especially in the cerebral cortex. Such a limitation would be expected to appear in the form of a trade-off between body mass and number of brain neurons, as seen in large non-human primates (Fonseca-Azevedo and Herculano-Houzel, 2012).

Carnivorans are divided into two main suborders, *Caniformia* and *Feliformia*, both of which include species that were domesticated, which has been suggested to alter the relationship between brain and body size (Kruska, 2007). In phylogenetic terms, carnivorans are closely related to artiodactyls (Bininda-Emonds et al., 2007), animals that the large meat-eating carnivorans prey upon. We have previously found that artiodactyls share with marsupials, afrotherians, glires, and eulipotyphlans the scaling relationship between number of cortical neurons and decreasing average neuronal density in the cerebral cortex (which reflects larger average neuronal cell sizes; Mota and Herculano-Houzel, 2014), such that the mass of the cerebral cortex scales faster than the cortex gains neurons across species (reviewed in Herculano-Houzel, 2016). Primates, on the other hand, have larger neuronal densities in the cerebral cortex than non-primates of similar cortical mass (Herculano-Houzel, 2016), and therefore larger numbers of neurons in similarly sized structures, which we have proposed to convey a cognitive advantage to primates (Herculano-Houzel, 2012). The relationship between body mass and number of brain neurons is highly variable in a clade-specific manner, but it is unlikely to contribute to cognitive capabilities across species (Herculano-Houzel, 2017). In contrast, all mammalian species examined so far share the same relationship between the mass of major brain structures and the numbers of non-neuronal cells that compose them (Herculano-Houzel, 2014; Dos Santos et al., 2017), which indicates that a single scaling rule has governed the addition of non-neuronal cells to brain tissue for at least 166 million years (Murphy et al., 2001, 2004; Bininda-Emonds et al., 2007).

Here we determine the cellular composition of the brain of eight carnivoran species (ferret, banded mongoose, raccoon, domestic cat, domestic dog, striped hyena, lion, and brown bear) to investigate several possibilities: (1) that all carnivoran brains and substructures follow the same non-neuronal scaling rules that apply to all other therians examined so far, with similar non-neuronal cell densities; (2) that different neuronal scaling rules apply to carnivoran brains compared to other non-primate brains, in particular such that carnivoran brains have more neurons than artiodactyl brains of similar mass; (3) that domesticated species diverge from wild species in their neuronal composition and relationship to body mass; and (4) that carnivoran brains exhibit evidence of an energetic trade-off between body mass and number of brain neurons, especially in the cerebral cortex.

## Materials and Methods

Here we use the isotropic fractionator (Herculano-Houzel and Lent, 2005; Herculano-Houzel, 2012) to determine the numbers of neuronal and non-neuronal cells that compose the main structures (olfactory bulb, hippocampus, cerebral cortex, cerebellum and rest of brain) of eight carnivoran species. The isotropic fractionator consists of dissolving brain tissue in a detergent solution to collect all cell nuclei in a suspension that can be made isotropic by agitation. Numbers of nuclei are determined by counting DAPI-stained samples under a fluorescent microscope; numbers of neurons are then calculated after determining the fraction of cell nuclei that express NeuN, a universal neuronal nuclear marker (Mullen et al., 1992). While some specific neuronal populations fail to express NeuN, such as mitral cells in the olfactory bulb and Purkinje cells in the hippocampus, those populations are negligible for the purpose of determining total numbers of neurons in the major brain structures and comparing them across species. Importantly, the isotropic fractionator has been shown to yield results that are comparable to those obtained with well-employed stereological techniques, and in less time (Herculano-Houzel et al., 2015b), which is fundamental for the analysis of large brains.

### Animals

**FIGURE 1:**
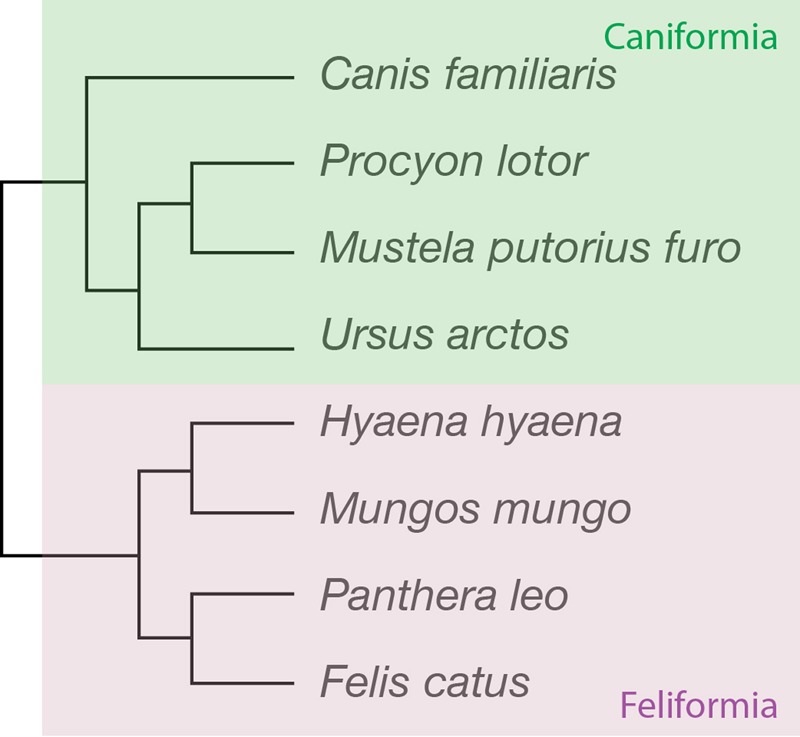
Phylogenetic relationships across the carnivoran species studied. Four of the species belong to the suborder *Caniformia* (dog, raccoon, ferret and brown bear), and four to the family *Feliformia* (striped hyena, banded mongoose, lion and cat).

We analyzed one brain hemisphere of one or two individuals of the following eight species: domestic ferret (*Mustela putorius furo, n* = 2), banded mongoose (*Mungos mungo, n* = 1), raccoon (*Procyon lotor, n* = 2), cat (*Felis catus, n* = 1), dog (*Canis familiaris, n* = 2), striped hyena (*Hyaena hyaena, n* = 1), African lion (*Panthera leo, n* = 1) and brown bear (*Ursus arctos, n* = 1). These species are divided into the suborders *Caniformia* (ferret, raccoon, dog, brown bear) and *Feliformia* (cat, banded mongoose, striped hyena, lion; **Figure 1**). Ferret, cat and dog individuals were bred in captivity, and are considered to represent domesticated species; the banded mongoose, African lion and brown bear specimens were obtained from the Copenhagen Zoo after being euthanized with sodium pentobarbital (i.v) in line with management decisions of the zoo; raccoons were wild caught in Cook County, IL, United States, with permission from The Cook County Forest Preserve Field Office in Chicago, IL, United States as part of their routine pathogen surveillance trapping; the striped hyena specimen was from an adult female that was obtained from the Saudi Wildlife Authority following veterinary euthanasia for unrelated medical reasons. Cat and dogs were donated by their owners after the natural death of the animals from non-neurological causes, with approval of the Federal University of Rio de Janeiro Committee for Ethics in the Use of Animals (process number 01200.001568/2013-87). The animals obtained from the Copenhagen Zoo and from Saudi Arabia were treated and used according to the guidelines of the University of Witwatersrand Animal Ethics Committee (clearance number 2012/53/1), which correspond with those of the NIH for care and use of animals in scientific experimentation. All other animals were killed by overdose with anesthetics according to NIH (ferrets, raccoons) and Brazilian (cats and dogs) veterinary guidelines. Once dead, the heads of the larger species were removed from the body and were perfused through the carotid arteries with a rinse of 0.9% saline (0.5 l/kg mass), followed by fixation with of 4% paraformaldehyde in 0.1 M phosphate buffer (PB) (1 l /kg mass) (Manger et al., 2009). Ferrets, mongoose and raccoons were perfusion fixed through the heart; striped hyena, cat and dog brains were only immersion-fixed once removed from the skull. All other brains were removed from the skull after perfusion and post-fixed (in 4% paraformaldehyde in 0.1 M PB) for 24–72 h at 4°C. The brains were then transferred to a solution of 30% sucrose in 0.1 M PB at 4°C until they had equilibrated and were then transferred to an antifreeze solution containing 30% glycerol, 30% ethylene glycol, 30% distilled water and 10% 0.244 M PB. Once again the brains were allowed to equilibrate in the solution at 4°C and were then moved to a -20°C freezer for storage prior to use in the current experiments. Cat and dog brains were fixed by immersion in 4% paraformaldehyde in 0.1M phosphate buffer for a total of approximately 2 weeks.

Brains were removed, cut into left and right halves, one of which was preserved for later histological studies, and the other used for quantification with the isotropic fractionator. When available (only for one raccoon and two ferrets), the olfactory bulb (OB) was first separated from the brain by transection immediately proximal to the main bulb. The cerebellum (CB) was dissected by cutting the peduncles at the surface of the brainstem. The brainstem was separated from the cerebrum by cutting along a plane anterior to the colliculi and posterior to the hypothalamus. The cerebrum was then cut into a series of 2 mm coronal sections, which were imaged on a flatbed scanner for subsequent morphometric analysis. From these sections, the hippocampus (HP) and the ensemble of diencephalon + striatum were removed, and the remaining cerebral cortex (CX) was then separated into gray and white matter components in each section. Although counted separately, in the present study we concern ourselves only with the totality of gray and white cortical matter, to which we also add the hippocampus (CxT), for the sake of comparison with previous studies on other mammalian species (data provided in Herculano-Houzel et al., 2015a). As in those studies, the ensemble of brainstem and diencephalon + striatum is reported here as the rest of brain (RoB), and whole brain (BR) refers to the sum of CxT, CB and RoB (that is, it excludes the olfactory bulb, for the sake of consistency, since the olfactory bulb is often not available for analysis; Herculano-Houzel et al., 2015a). Each structure was weighed prior to homogenization. All values in tables and graphs correspond to masses and cell number estimates (or averages, where two individuals were available) multiplied by two, to represent both sides of the brain, as in our previous studies (Herculano-Houzel et al., 2015a). This procedure assumes that any differences between two sides of the brain are negligible compared to the differences across species that span several orders of magnitude in brain structure mass and numbers of cells in the present study.

### Morphometry

Images of all coronal sections of the cerebral cortex were imported into StereoInvestigator software (MBF Bioscience, Williston, VT, United States) for tracing and reconstruction of total and exposed cortical surface areas, and for Cavalieri analysis to determine gray and white matter volumes, using formulas described previously (Ribeiro et al., 2013; Kazu et al., 2014). The average thickness of the gray matter was calculated as the ratio between total gray matter volume and surface area, and the folding index of the whole cortex was calculated as the ratio between total gray matter surface area and exposed cortical surface area, as in a previous study of cortical folding (Mota and Herculano-Houzel, 2015).

### Isotropic Fractionator

After weighing, each structure was sliced by hand and dissolved in a solution of 1% Triton X-100 in 30 mM sodium citrate in a glass Tenbroeck homogenizer (Pyrex, Corning, NY, United States) until no visible particles remained. The homogenate and several washes of the homogenizer were collected in a graduated cylinder to which DAPI (4,6-diamidino-2-phenylindole, Invitrogen, United States) was added in a dilution of 1:20 to 1:50 from a stock solution of 20 mg/l. The volume of the suspension was rounded up with PBS to a value that could be read with precision on the graduated cylinder. After agitating the suspension, with care not to form bubbles, typically four aliquots were taken and placed on Neubauer improved chambers for counting under a fluorescent microscope (Zeiss, Jena, Germany). Typically, numbers of nuclei were counted in volumes of either 40 or 100 nl on the chambers, whichever sufficed to ensure that at least 60 but not more than 300 nuclei were counted per aliquot. Four aliquots were considered sufficient for a reliable estimate when they yielded a coefficient of variation (CV) of less than 0.15. Typically, CVs were well below 0.10. Isotropic fractionation thus provides estimates of numbers of cells that are at least as reliable as those obtained with stereology (Herculano-Houzel et al., 2015b). Importantly, the small CVs mean that our estimates of cell numbers have standard deviations of less than 10% of the estimate for each specimen, in contrast to the variation of orders of magnitude across species, which is crucial given that we often have only one specimen of each species available for analysis.

A sample of 500 μl of each suspension was then washed in phosphate-buffered saline (PBS) and reacted overnight with Cy3-conjugated rabbit polyclonal anti-NeuN antibody (ABN78C3, Millipore, United States) at room temperature. The next day, samples were washed and resuspended in PBS, and stained again with a 1:20–1:50 dilution of the stock solution of DAPI. One aliquot of each sample was then inspected under the fluorescence microscope for counting the fraction of at least 500 DAPI-labeled nuclei that also exhibited NeuN immunoreactivity. This fraction was multiplied by the total number of nuclei (and therefore cells) previously obtained in that structure to yield the total number of neurons. This procedure was followed for all species except for the hyena, whose brain had been fixed in paraformaldehyde too long to allow immunohistochemistry. In this species, the fraction of neuronal nuclei was determined according to morphological criteria: nuclei were considered to belong to neurons if they were round (independent of size), exhibited loose chromatin and a single nucleolus. The number of non-neuronal cells was obtained by subtracting the number of neurons from the total number of cells. Densities of neuronal and non-neuronal cells correspond to the number of the respective cells in the structure divided by the mass of the structure in milligrams (cells/mg).

### Mathematical Analyses

All analyses were performed in JMP 9.0 (SAS, Cary, NC, United States). Power functions were calculated by fitting a linear function to log-transformed data using least-squares regression. All analyses are performed separately for each mammalian clade; clades are pooled only when regression analyses show that scaling relationships are described by similar functions. Values obtained for carnivoran species (*n* = 8) were compared to those predicted for other non-primate mammalian species in our dataset (*n* = 37) to test whether carnivorans conform to the non-primate mammalian scaling rules for different brain structures in relation to numbers of cells and to body mass. The role of phylogenetic clustering is examined directly by performing each analysis separately by clade. We do not use methods to account for phylogenetic relatedness *within* each clade because our main focus is on scaling relationships between numbers of cells, cell density and structure mass as well as their absolute numbers in key species, irrespective of any presumed phylogenetic relationships among them. All raw data are provided so that those interested in testing these relationships within clades may do so.

## Results

**FIGURE 2:**
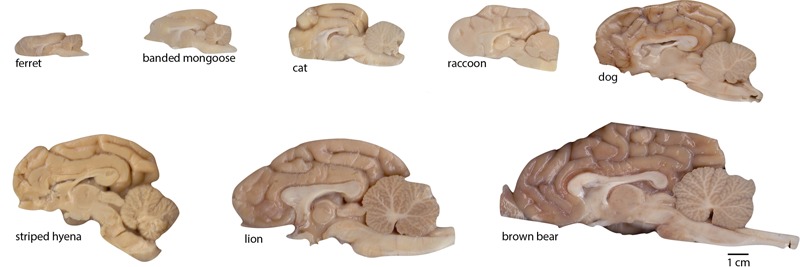
Examples of brain hemispheres of the carnivoran species studied. Images show the medial aspect of one half-brain (not necessarily the right half as the images suggest; some are mirror-images for conformity). All images are shown to the same scale (scale bar, 1 cm).

In our sample of carnivoran species, body mass varied 437.5-fold between ferret and brown bear, the smallest and largest species examined, whereas brain mass varied only 58.0-fold, and the total number of neurons in the brain only 23.7-fold, between the same two species (**Figure 2** and **Table 1**). The discrepancy between the large variation in body mass and number of brain neurons is consistent with the trend that we have revealed of much faster increases in body mass than in numbers of brain neurons (Herculano-Houzel, 2017). Moreover, the larger increase in brain mass than in number of neurons across species is a first indication that larger brains have more but also bigger neurons, as found in non-primate species (Herculano-Houzel et al., 2014b). Data are summarized in **Table 1**.

**Table 1:** Summary of the cellular composition of the major brain structures of eight carnivoran species.

	Ferret	Banded mongoose	Raccoon	Cat	Dog	Striped hyena	Lion	Brown bear
	*(Mustela putorius furo)*	*(Mungos mungo)*	*(Procyon lotor)*	*(Felis catus)*	*(Canis familiaris)*	*(Hyaena hyaena)*	*(Panthera leo)*	*(Ursus arctos)*
M_BD_, kg	0.8	1.5	6.2	4.5	19.7	40	180	350
M_BR_, g	5.429	14.292	34.186	34.858	86.462	99.214	198.946	315.004
M_CxT_, g	3.123	9.290	24.491	24.176	65.491	67.450	139.902	222.000
M_HP_, g	0.329	0.428	0.477	0.876	2.010	3.366	3.648	4.130
M_CB_, g	0.920	1.548	3.338	5.110	7.512	14.032	24.882	45.106
M_RoB_, g	1.386	3.454	6.357	5.572	13.459	17.732	34.162	47.898
M_OB_, g	0.25	n.a.	0.47	n.a.	n.a.	n.a.	n.a.	n.a.
N_BR_	404.43 × 10^6^	454.02 × 10^6^	2,148.07 × 10^6^	1,215.21 × 10^6^	2,252.69 × 10^6^	3,884.60 × 10^6^	4,667.03 × 10^6^	9,585.60 × 10^6^
N_CxT_	38.95 × 10^6^	115.77 × 10^6^	437.94 × 10^6^	249.83 × 10^6^	527.91 × 10^6^	495.28 × 10^6^	545.24 × 10^6^	250.97 × 10^6^
N_HP_	3.19 × 10^6^	2.66 × 10^6^	15.34 × 10^6^	8.05 × 10^6^	8.61 × 10^6^	18.24 × 10^6^	13.54 × 10^6^	8.61 × 10^6^
N_CB_	351.26 × 10^6^	315.74 × 10^6^	1,646.18 × 10^6^	942.90 × 10^6^	1,676.63 × 10^6^	3,333.83 × 10^6^	4,049.75 × 10^6^	9,253.25 × 10^6^
N_RoB_	14.21 × 10^6^	22.51 × 10^6^	48.57 × 10^6^	22.47 × 10^6^	48.15 × 10^6^	55.49 × 10^6^	72.04 × 10^6^	81.38 × 10^6^
N_OB_	14.04 × 10^6^	n.a.	55.99 × 10^6^	n.a.	n.a.	n.a.	n.a.	n.a.
O_BR_	472.98 × 10^6^	652.37 × 10^6^	2,399.99 × 10^6^	1,728.79 × 10^6^	5,193.23 × 10^6^	4,501.39 × 10^6^	12,398.84 × 10^6^	15,113.19 × 10^6^
O_CxT_	264.06 × 10^6^	346.56 × 10^6^	1,766.67 × 10^6^	1,097.10 × 10^6^	3,416.46 × 10^6^	2,977.76 × 10^6^	7,260.97 × 10^6^	10,154.00 × 10^6^
O_HP_	29.31 × 10^6^	22.96 × 10^6^	70.00 × 10^6^	55.95 × 10^6^	134.05 × 10^6^	139.82 × 10^6^	177.11 × 10^6^	249.16 × 10^6^
O_CB_	79.60 × 10^6^	154.26 × 10^6^	241.32 × 10^6^	326.10 × 10^6^	848.68 × 10^6^	594.29 × 10^6^	3,044.00 × 10^6^	2,296.75 × 10^6^
O_RoB_	129.33 × 10^6^	151.54 × 10^6^	392.00 × 10^6^	305.59 × 10^6^	928.09 × 10^6^	929.34 × 10^6^	2,093.86 × 10^6^	2,662.44 × 10^6^
O_OB_	43.48 × 10^6^	n.a.	79.76 × 10^6^	n.a.	n.a.	n.a.	n.a.	n.a.
DN_CxT_	12,473	12,462	18,510	10,334	8,061	7,343	3,897	1,130
DN_HP_	9,702	6,225	16,076	9,187	4,282	5,418	3,711	2,085
DN_CB_	381,810	203,966	493,162	184,521	223,194	237,588	162,758	205,145
DN_RoB_	10,253	6,518	7,640	4,033	3,577	3,129	2,109	1,699
DN_OB_	56,150	n.a.	119,119	n.a.	n.a.	n.a.	n.a.	n.a.
O/N_BR_	1.253	1.437	1.117	1.423	2.305	1.159	2.657	1.577
O/N_CXT_	6.779	2.994	3.897	4.391	6.472	6.012	13.317	40.459
O/N_HP_	9.182	8.618	4.564	6.952	15.575	7.667	13.083	28.941
O/N_CB_	0.227	0.489	0.147	0.346	0.506	0.178	0.752	0.248
O/N_RoB_	9.101	6.731	8.071	13.598	19.275	16.749	29.063	32.714
O/N_OB_	3.097	n.a.	1.425	n.a.	n.a.	n.a.	n.a.	n.a.

*Species are ordered according to brain mass. M, mass of body (M_*BD*_) or brain structure; N, number of neurons; O, number of other (non-neuronal) cells; DN, neuronal density (in neurons/mg); O/N, ratio between numbers of other (non-neuronal) cells and neurons. BR, whole brain (excluding the olfactory bulb); CXT, whole cerebral cortex (gray matter, white matter and hippocampus); HP, hippocampus; CB, cerebellum; RoB, rest of brain (the sum of diencephalon + basal ganglia, mesencephalon, and pons+medulla); OB, olfactory bulb. All values refer to the two hemispheres together, calculated as 2 × values for one hemisphere.*

**FIGURE 3:**
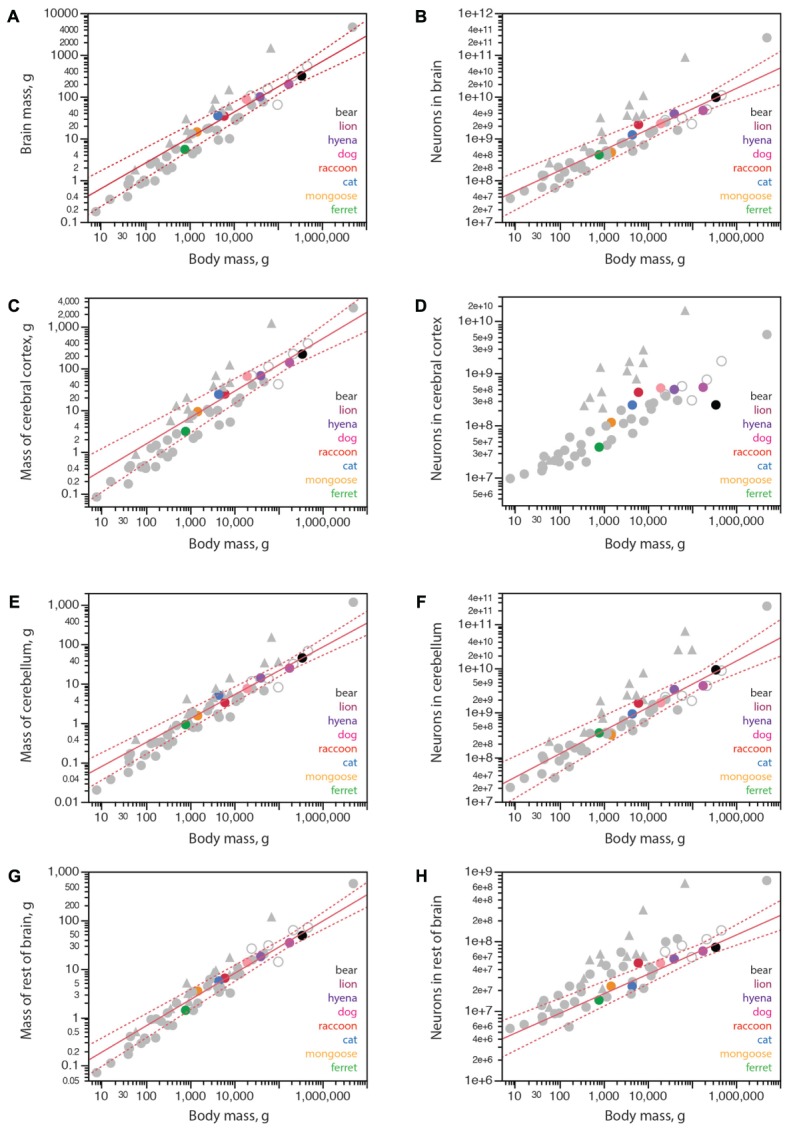
Structure mass and number of neurons scale with body mass across carnivoran species, except for the number of neurons in the cerebral cortex. Plotted functions, in red, apply to carnivoran species and include the 95% confidence interval for the fit. Carnivoran species analyzed in this study are shown in colors according to the key in the graphs; non-carnivoran species are depicted in gray (primates in triangles, artiodactyls as unfilled circles). **(A)** Brain mass scales as a power function of body mass with exponent 0.608 ± 0.051 across carnivoran species (*r*^2^ = 0.959, *p* < 0.0001, *n* = 8, plotted), which is not significantly different from the exponent of 0.548 ± 0.038 that applies across artiodactyls (*r*^2^ = 0.990, *p* = 0.0048, *n* = 4), but is significantly lower than the exponent that applies across primates (0.903 ± 0.082, *r*^2^ = 0.931, *p* < 0.0001, *n* = 11). **(B)** The number of neurons in the brain scales as a power function of body mass with exponent 0.492 ± 0.054 across carnivoran species (*r*^2^ = 0.932, *p* = 0.0001, *n* = 8, plotted), which is indistinguishable from the exponent of 0.448 ± 0.115 that applies across artiodactyls (*r*^2^ = 0.884, *p* = 0.0598, *n* = 4), but is significantly lower than the exponent of 0.777 ± 0.091 (*r*^2^ = 0.889, *p* < 0.0001, *n* = 11) that applies across primates. **(C)** The mass of the cerebral cortex of carnivorans scales as a power function of body mass with exponent 0.631 ± 0.062 (*r*^2^ = 0.944, *p* < 0.0001, *n* = 8, plotted), which is not significantly different from the exponent that applies across artiodactyl species (0.589 ± 0.028, *r*^2^ = 0.995, *p* = 0.0023, *n* = 4), although smaller than the exponent that applies across primate species (0.942 ± 0.084, *r*^2^ = 0.926, *p* < 0.0001). **(D)** Whereas larger body mass is accompanied by larger numbers of cortical neurons in all other mammalian clades examined, larger carnivorans do not have ever growing numbers of cortical neurons. The lion and striped hyena have only as many cortical neurons as the average dog, which is only slightly more neurons than the raccoon, and the brown bear has even fewer cortical neurons, about as many as found in the cat. **(E)** The mass of the cerebellum scales as a power function of body mass with exponent 0.606 ± 0.042 across carnivorans (*r*^2^ = 0.972, *p* < 0.0001, *n* = 8, plotted), which is undistinguishable from artiodactyls (exponent, 0.612 ± 0.105, *r*^2^ = 0.944, *p* = 0.0282, *n* = 4), but lower than the exponent of 0.739 ± 0.074 (*r*^2^ = 0.900, *p* < 0.0001, *n* = 12) that applies to primates. **(F)** The number of neurons in the cerebellum scales as a power function of body mass with exponent 0.522 ± 0.056 (*r*^2^ = 0.935, *p* < 0.0001, *n* = 8, plotted), which overlaps with the power functions that apply to other clades except for primates (exponent, 0.754 ± 0.073, *r*^2^ = 0.906, *p* < 0.0001, *n* = 13) and for eulipotyphlans (exponent, 0.873 ± 0.088, *r*^2^ = 0.970, *p* = 0.0022, *n* = 5). **(G)** The mass of the rest of brain of carnivoran species scales as a power function of body mass with exponent 0.540 ± 0.035 (*r*^2^ = 0.976, *p* < 0.0001, *n* = 8, plotted), which overlaps with the power functions that apply to other clades except for primates (exponent, 0.706 ± 0.076, *r*^2^ = 0.896, *p* < 0.0001, *n* = 12). **(H)** The number of neurons in the rest of brain of carnivoran species scales as a power function of body mass with exponent 0.269 ± 0.042 (*r*^2^ = 0.872, *p* = 0.0007, *n* = 8), or 0.282 ± 0.028 without the raccoon (*r*^2^ = 0.953, *p* = 0.0002, *n* = 7, plotted), which has more neurons in the rest of brain than expected for body mass. Although the first exponent is not significantly different from the exponent that applies to artiodactyls (*r*^2^ = 0.227 ± 0.027, *r*^2^ = 0.973, *p* = 0.0136, *n* = 4), carnivoran species seem to have fewer neurons in the rest of brain than artiodactyls of similar body mass (unfilled circles).

Carnivorans have brain masses in the same range as other non-primate mammals of similar body mass, including artiodactyls, although the power function that relates brain mass to body mass has a smaller exponent of 0.608 ± 0.051 in carnivorans (**Figure 3A**) compared to 0.712 ± 0.071 in glires, 0.742 ± 0.061 in marsupials, 0.903 ± 0.082 in primates (all exponents have *p*-values < 0.0001), but is not significantly different from the exponent of 0.548 ± 0.038 (*p* = 0.0048) in artiodactyls (unfilled symbols in **Figure 3A**). Notice that although the exponent that relates brain mass to body mass across carnivoran species is not significantly different from the exponent that applies to artiodactyls, carnivorans, lion and hyena in particular, have slightly smaller brains than artiodactyl species of similar body mass (**Figure 3A**; the outlier artiodactyl in the figure is the domesticated pig, which has a very large body mass for its brain mass). Still, carnivoran brains have numbers of neurons comparable to those found in non-primate mammals of similar body mass, in particular artiodactyls, although clade-specific exponents are again observed (**Figure 3B**). For instance, the lion and hyena, at a total 3.9–4.7 billion neurons, have the same range of brain neurons as the blesbok and greater kudu, at 3.0–4.9 billion neurons (Herculano-Houzel et al., 2015a).

Within the brain, carnivorans have cerebella of comparable mass and numbers of neurons to other non-primate mammals of similar body mass, artiodactyls in particular (**Figures 3E,F**). Interestingly, the rest of brain is somewhat smaller in mass in carnivorans compared to artiodactyls of similar body mass (**Figure 3G**), and also has significantly fewer neurons in carnivorans compared to artiodactyls and actually several other non-primate mammalian species of similar body mass (**Figure 3H**). The raccoon, however, appears to have more neurons in the rest of brain than predicted for its body mass; indeed, removing the raccoon from the analysis improves the fit of the function that describes how the number of neurons in the RoB scales with body mass across carnivoran species (**Figure 3H**).

As found in other mammalian clades, larger carnivoran species have larger cerebral cortices, whose mass is comparable to that of artiodactyls and several other non-primate mammalian species of similar body mass (**Figure 3C**). Strikingly, however, larger carnivorans do not have increasingly more neurons in the cerebral cortex. While ferret, mongoose and cat have increasingly larger cortices (3.1 g, 9.3 g, and 24.2 g) with increasingly more neurons (39 million, 116 million, and 250 million neurons, respectively), we find that the lion has approximately as many neurons in the cerebral cortex as the average found in dogs, ca. 500 million neurons, despite a twice larger cortex in the lion than in the dogs (139.9 g vs. 65.5 g; **Table 1**). Even more strikingly, the brown bear has fewer neurons in the cerebral cortex than these two species, 251 million neurons, which is only about as many as the house cat, even though the brown bear cortex had a nearly 10-fold larger mass of 222.0 g (**Figure 3D** and **Table 1**). The raccoon also stands out in its number of cortical neurons, but in a different direction: although the mass of the cerebral cortex in both raccoon and cat is a similar 24 g, the raccoon cerebral cortex has an average 438 million neurons compared to 250 million neurons in the cat (**Table 1**). Remarkably, of all the individuals we analyzed, the one with the most neurons in the cerebral cortex was a golden retriever dog (627 million neurons), followed by the lion (545 million neurons), one of the raccoons (512 million neurons), the striped hyena (495 million neurons), a smaller dog of unspecified breed (429 million neurons) and a second raccoon individual (395 million neurons). As a result, the relationship between numbers of cerebral cortical neurons and body mass in carnivorans seems to saturate around 500–600 million neurons, and possibly adopt the shape of an inverted U with only half as many neurons in the brown bear cerebral cortex (**Figure 3D**), a pattern that has not been observed in any other mammalian clade so far, where simple power laws apply (Herculano-Houzel et al., 2014b).

**FIGURE 4:**
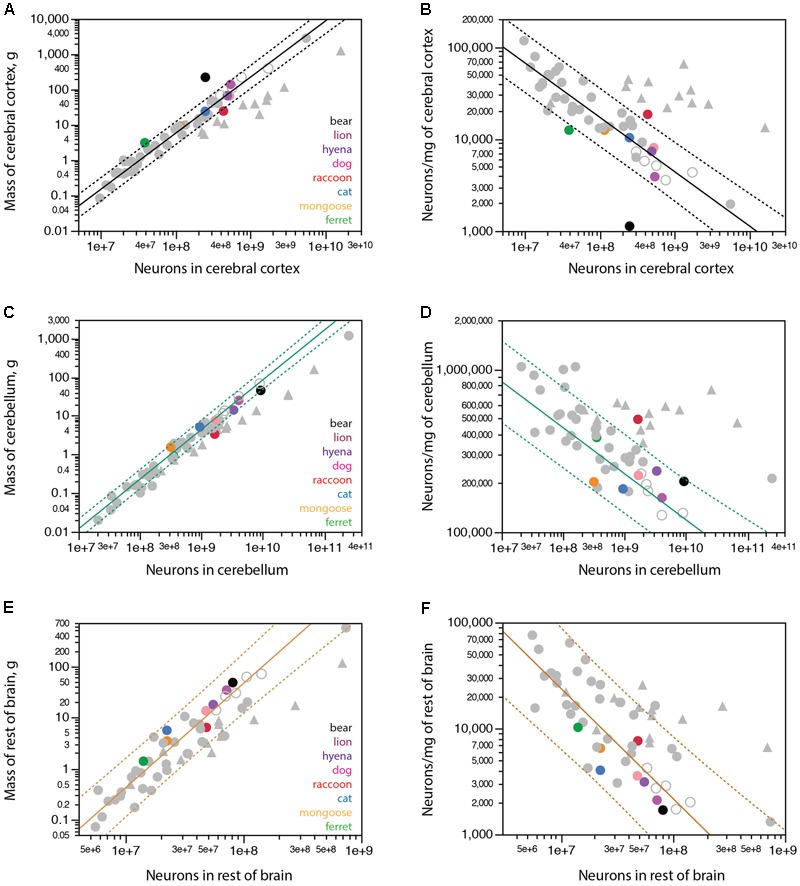
Scaling of mass of brain structures with numbers of neurons in carnivorans. Carnivoran species analyzed in this study are shown in colors according to the key in the graphs; non-carnivoran species are depicted in gray (primates in triangles, artiodactyls as unfilled circles). Plotted functions apply to the species indicated for each graph and include the 95% confidence interval for individual values. **(A)** With the exception of the brown bear, ferret and raccoon, carnivoran species conform to the power function that describes how the mass of the cerebral cortex scales as a power function of the number of cortical neurons with exponent 1.588 ± 0.042 across non-primate, non-carnivoran species (*r*^2^ = 0.978, *p* < 0.0001, *n* = 35, plotted). The function calculated for carnivorans (without the bear and raccoon) has an exponent of 1.311 ± 0.136 (*r*^2^ = 0.959, *p* = 0.0006, *n* = 6). **(B)** Again, with the exception of the brown bear, ferret and raccoon, the density of neurons in the cerebral cortex of carnivoran species conforms to the power function that describes the scaling of neuronal density with the number of cortical neurons of exponent –0.590 ± 0.040 across non-primate, non-carnivoran species (*r*^2^ = 0.865, *p* < 0.0001, *n* = 35, plotted). **(C)** With the exception of the raccoon, carnivoran species conform to the power function that describes how the mass of the cerebellum scales as a power function of the number of cerebellar neurons with exponent 1.283 ± 0.035 across the ensemble of afrotherians (minus the African elephant), artiodactyls and glires (*r*^2^ = 0.987, *p* < 0.0001, *n* = 20, plotted). **(D)** With the exception of the raccoon, the density of neurons in the cerebellum of carnivoran species conforms to the power function that describes the scaling of neuronal density with the number of cerebellar neurons of exponent –0.283 ± 0.035 across the ensemble of afrotherians (minus the African elephant), artiodactyls and glires (*r*^2^ = 0.784, *p* < 0.0001, *n* = 20, plotted). However, the power function calculated for carnivoran species (minus the raccoon) fails to reach significance (*p* = 0.2918). **(E)** The power function that describes how the mass of the rest of brain scales with the number of rest of brain neurons across artiodactyls (minus the giraffe), eulipotyphlans and marsupials (exponent, 2.041 ± 0.143, *r*^2^ = 0.928, *p* < 0.0001; plotted) includes carnivoran species. **(F)** Carnivorans are aligned with the scaling of neuronal density in the rest of brain with the number of rest of brain neurons that applies to the ensemble of artiodactyls (minus the giraffe), eulipotyphlans and marsupials, with exponent –1.040 ± 0.142 (*r*^2^ = 0.769, *p* < 0.0001, *n* = 18, plotted).

In line with the smaller numbers of cortical neurons than expected for the mass of the cerebral cortex in the largest carnivorans examined, we find that while the banded mongoose, cat, dog and hyena conform to the scaling relationship between cerebral cortical mass and numbers of cortical neurons that applies to non-primate mammals (**Figure 4A**, plotted function), the lion has fewer neurons in its cerebral cortex than expected for its cortical mass (although still within the 95% confidence interval for individual values), and the brown bear falls well outside the 95% confidence interval for that relationship, with about aaa the number of neurons predicted for its cortical mass (**Figure 4A**, black). The ferret also has fewer neurons in the cerebral cortex than expected for a non-primate, although close to the 95% confidence interval (**Figure 4A**, green). In contrast, the raccoon has more neurons in its cerebral cortex than expected for a non-primate mammal of its cortical mass, approaching the relationship expected for a primate (**Figure 4A**, red circle and triangles). Indeed, while the banded mongoose, cat, dog and hyena have neuronal densities in the cerebral cortex that decrease predictably with increasing numbers of cortical neurons according to the scaling relationship that applies to other non-primates (**Figure 4B**), the raccoon has an average neuronal density in the cerebral cortex that is about three times the expected for a non-primate mammal with its number of neurons in the cerebral cortex, approaching neuronal densities found in primate cortices. On the other hand, neuronal densities are several times smaller than expected in the ferret, lion and especially the brown bear cerebral cortex for their numbers of cortical neurons, compared to non-primate mammals (**Figure 4B**).

In contrast to the cerebral cortex, the cerebellum of all carnivorans in the dataset conforms to the neuronal scaling rule that applies to the ensemble of afrotherians (minus the elephant), glires, and artiodactyls – with the sole exception, again, of the raccoon (**Figure 4C**). The relationship between cerebellar mass and number of cerebellar neurons of carnivorans (excluding the raccoon) can be described by a power law of exponent 1.100 ± 0.084 (*r*^2^ = 0.971, *p* < 0.0001) that is not significantly different from linearity but is significantly different from the exponent of 1.283 ± 0.035 that applies to the ensemble of afrotherians (minus the elephant), glires and artiodactyls, which we have proposed to represent the ancestral neuronal scaling rule for the mammalian cerebellum (Herculano-Houzel et al., 2014b). In contrast, the raccoon cerebellum has nearly two times more neurons than predicted for a mammalian species belonging to those non-primate orders, conforming instead to the number of neurons found in the cerebellum of a primate of similar cerebellar mass. As expected from these relationships, neuronal densities in the cerebellum of carnivorans, again with the exception of the raccoon, conform to the relationship that applies to the ensemble of afrotherians (minus the elephant), glires and artiodactyls (**Figure 4D**), even though the power function relating neuronal densities in the cerebellum of carnivorans to the number of neurons in the cerebellum does not reach significance (*p* = 0.2918 without the raccoon; **Figure 4D**).

The mass of the carnivoran rest of brain scales with the number of neurons in the structure raised to an exponent of 1.875 ± 0.134 (without the raccoon). This exponent is not significantly different from the exponent of 2.041 ± 0.143 that applies to the ensemble of artiodactyls, marsupials and eulipotyphlans (*r*^2^ = 0.928, *p* < 0.0001; **Figure 4E**; Herculano-Houzel, 2017), and indeed the 95% confidence interval for carnivorans includes these species (**Figure 4E**). Rodents, afrotherians and primates depart from this relationship (**Figure 4E**). Carnivoran species conform to the relationship that describes how neuronal density in the RoB decreases with increasing number of neurons in the RoB across artiodactyls, eulipotyphlans and marsupials (**Figure 4F**).

### Other Cells

**FIGURE 5:**
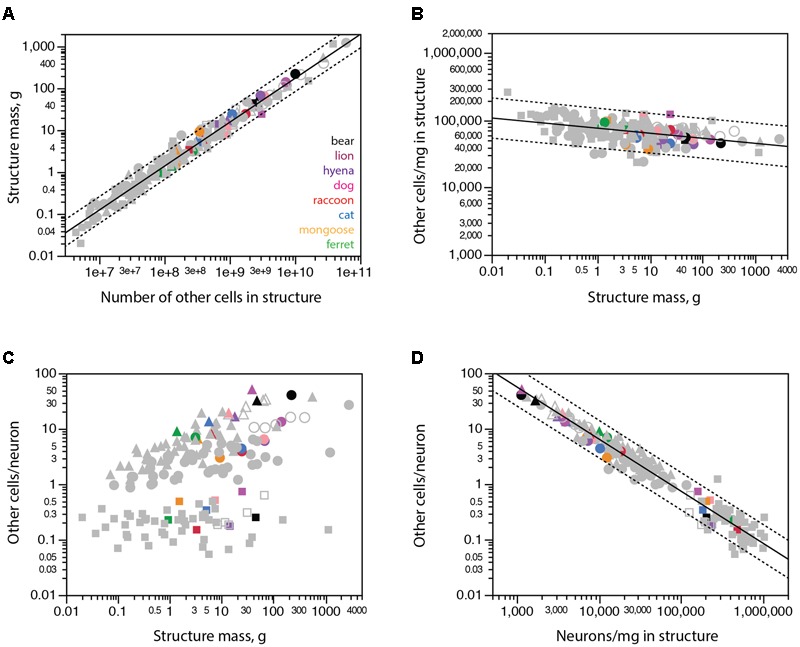
All carnivoran species and brain structures conform to the scaling of brain structure mass with numbers of other cells that applies universally across other mammalian species. Cerebral cortex is shown in circles, cerebellum in squares, rest of brain in triangles. **(A)** Brain structure mass scales universally as a power function of the number of non-neuronal (other) cells in the structures across non-carnivoran species (plotted function; exponent 1.051 ± 0.014, *r*^2^ = 0.974, *p* < 0.0001), and all carnivorans conform to that relationship. **(B)** The density of other cells in the different structures of carnivoran brains overlaps with densities in the same structures in other mammalian species, which scales very slowly as a power function of structure mass of exponent -0.075 ± 0.012 (*r*^2^ = 0.200, *p* < 0.0001). **(C)** The ratio between numbers of other cells (which approximates the number of glial cells) and numbers of neurons in each structure is not a universal function of structure mass across mammalian species and structures. **(D)** The ratio between numbers of other cells and neurons in each structure does vary universally with average neuronal density in the structure across non-carnivoran species (plotted function, exponent -0.942 ± 0.019, *r*^2^ = 0.946, *p* < 0.0001, *n* = 146), and all carnivoran species and brain structures conform to that relationship.

The discrepancies between expected and observed numbers of neurons in some carnivoran species, most notably the brown bear, could in principle be due to aberrant immunoreactivity to NeuN, which might fail to label all neurons in these species. Similarly, the aberrantly large numbers of neurons found in raccoon brain structures might in principle be due to non-specific labeling of non-neuronal cells with the anti-NeuN antibody. In these scenarios, any unlabeled neurons in the bear cerebral cortex would be classified as non-neuronal cells and cause aberrantly high numbers of non-neuronal cells in brain structures for their mass; conversely, any labeled glial cells would be mistakenly classified as neurons and lead to aberrantly low numbers of non-neuronal cells in raccoon brain structures. These aberrations would be particularly easy to spot since all major brain structures (cerebral cortex, cerebellum and rest of brain) of all mammalian species examined so far exhibit a relationship between structure mass and number of other (non-neuronal) cells that can be described by a single power function of near-linear exponent 1.051 ± 0.014 (*r*^2^ = 0.974, *p* < 0.0001; **Figure 5A**; Herculano-Houzel, 2017).

Instead, we find that all carnivoran species and brain structures analyzed conform to the relationship that applies to all other mammalian species, including all raccoon brain structures and the brown bear cerebral cortex (**Figures 5A,B**, colored points). The conformity of carnivoran data to the relationship that applies to all other mammalian species and brain structures confirms the universality of the non-neuronal scaling rules (Herculano-Houzel, 2014; Mota and Herculano-Houzel, 2014). This conformity also makes it highly unlikely that the unexpectedly small (or large) numbers of neurons in the brown bear cerebral cortex (or raccoon brain structures) are due to misclassification of cells as neurons.

As shown before (Herculano-Houzel, 2014), the ratio between numbers of other cells and neurons is not a universal function of structure mass across mammalian species, including carnivorans (**Figure 5C**). However, this ratio does scale universally with neuronal density in the structure across all mammalian species analyzed so far, and all carnivorans studied here, including the raccoon and brown bear, conform to that same relationship (**Figure 5D**).

### Domesticated vs. Wild Species

The dogs and cat individuals analyzed in this study were domesticated animals, raised by families who donated the brains after the animals died of natural causes, in contrast to other animals that were either wild-caught (raccoon, hyena) or kept in captivity (which might lead to larger body mass, but are expected to be representative of wild species). Notably, we find that these domesticated animals do not deviate from the relationship between brain mass (or number of neurons) and body mass that applies to carnivorans or to non-primates as a whole (**Figure 3**). Additionally, cat and dog data points conform to the relationships between brain structure mass and number of neurons in the structure that apply to other carnivoran as well as various non-primate clades (see **Figure 4**). Both dog individuals examined (a 7.45 kg mixed-breed and a 32 kg golden retriever) had larger brains than the cat (brain mass in dogs, 58.4 and 114.5 g, respectively; cat, 34.8 g), and also more brain neurons than the cat (dogs, 1.8 and 2.6 billion neurons, respectively; cat, 1.2 billion neurons). The same applies to the cerebral cortex of the dogs, at 46.2 g with 429 million neurons and 84.8 g with 623 million neurons, against 24.2 g with 250 million neurons in the cat. Strikingly, although the cerebral cortex of the golden retriever was almost twice as large as the cortex of the smaller dog, it only had 46% more neurons than the smaller dog cortex (as expected from the non-linear scaling of cortical mass with number of cortical neurons, **Figure 4**); if plotted separately, both individuals conform to the scaling rules that apply to carnivoran species shown in **Figure 4**, and as expected from their larger cortical mass, both dogs had more neurons in the cerebral cortex than the cat. Thus, the two most common species of domesticated carnivorans do not deviate from the relationship between cortical mass and number of neurons that applies both to wild carnivorans and other non-primate species of similar body, brain or cerebral cortical mass.

### Distribution of Cortical Neurons into Cortical Surface Area and Thickness

The apparently decreased number of neurons in the cerebral cortex of large carnivorans for their cortical and body mass, notably in the brown bear, could in principle be the result of altered development that led to the generation of smaller numbers of much larger neurons, resulting in the observed lower neuronal densities but expected non-neuronal densities. In that case, we might expect the cortical volume to still be distributed into surface area and thickness following the same scaling relationship that applies to other carnivorans, with larger surface areas accompanied by slowly increasing cortical thickness. Alternatively, if the unexpectedly small number of neurons in the cerebral cortex of large carnivorans is due to regressive phenomena after the cortex develops, such as pronounced neuronal loss after cortical expansion, we should find evidence of atrophy in the cerebral cortex of these species, with cortical thinning for their surface area, and possibly also a thicker cortex for their numbers of cortical neurons (in case the maximal attained thickness is not entirely lost), compared to the allometric scaling that applies to other carnivoran species (but still the same expected non-neuronal densities). We thus determined how the cortical volume was distributed into surface area and thickness across carnivoran species, and how that distribution related to numbers of cortical neurons.

**FIGURE 6:**
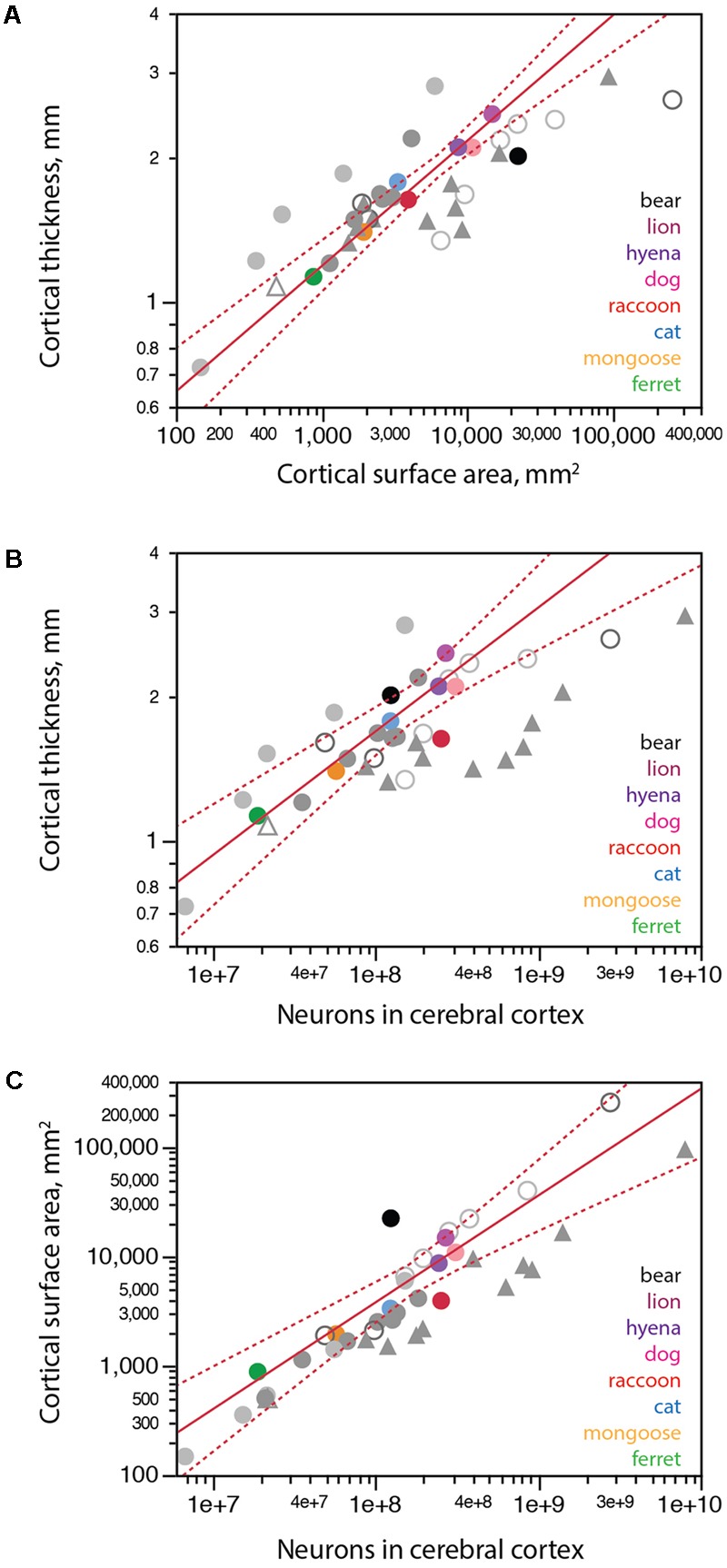
Scaling of cortical surface area and thickness with number of neurons in carnivorans. Each carnivoran species is shown in a different color according to the key in the graphs. All other mammals are depicted in gray (light gray filled circles, glires; light gray unfilled circles, artiodactyls; dark gray filled circles, marsupials; dark gray unfilled circles, afrotherians; filled triangles, primates; white triangle, scandentia). For the sake of clarity, scaling relationships for non-carnivoran clades are not plotted. **(A)** Average cortical thickness scales as a power function of cortical surface area with exponent 0.262 ± 0.021 (*r*^2^ = 0.969, *p* < 0.0001, *n* = 7, excluding the brown bear; plotted in red; excluding further the raccoon does not change the exponent, which remains 0.262 ± 0.022, *p* = 0.0003). The brown bear has a much thinner cortex for its surface area compared to other carnivoran species; the raccoon, in contrast, has the predicted combination of cortical surface area and thickness for a carnivoran. **(B)** Average cortical thickness scales as a power function of the number of cortical neurons with exponent 0.259 ± 0.031 (*r*^2^ = 0.945, *p* = 0.0011, *n* = 6, excluding the brown bear and raccoon; plotted in red). **(C)** Cortical surface area scales as a power function of the number of cortical neurons with exponent 0.978 ± 0.115, undistinguishable from linearity (*r*^2^ = 0.948, *p* = 0.0010, *n* = 6, excluding the brown bear and raccoon, plotted in red). All values refer to a single cortical hemisphere.

We find that carnivoran cerebral cortices with larger surface areas are also thicker, although cortical thickness increases more slowly than surface area, as a power function of surface area with exponent 0.262 ± 0.021 across carnivoran species (excluding the brown bear; **Figure 6A**). While the raccoon and lion have combinations of cortical surface area and thickness that match the prediction for carnivoran species, the brown bear is a clear outlier, with a cortical thickness that is too small for its surface area, suggestive of cortical atrophy (thinning).

The distribution of cortical neurons into surface area and thickness also suggest that a regressive phenomenon is in place. Both the brown bear and lion cortices are thicker than expected for the number of neurons in the cerebral cortex (**Figure 6B**, black), consistent with cortices that had more neurons in early development, attained adult-like morphology, but then lost significant numbers of neurons (and along with them, lost part of the width of the parenchyma, but not all of it). Similarly, the surface area of the brown bear cerebral cortex is almost one order of magnitude larger than expected for its number of neurons (**Figure 6C**, black). This pattern is consistent with a reduction in number of neurons in the cerebral cortex that occurred after cortical expansion in development, when the brain attained its adult density of non-neuronal cells, volume and surface area, leading to partial thinning of the cerebral cortex but very little loss of surface area.

In contrast to the bear, the raccoon has many more neurons than predicted for a carnivoran species with either its cortical thickness (**Figure 6B**, red) or its cortical surface area (**Figure 6C**, red), even though its cortical thickness x surface area relationship conforms to the pattern that applies to other carnivoran species (excluding the brown bear; **Figure 6A**). The larger than expected number of cortical neurons in the presence of the clade-typical relationship between cortical thickness and surface area is consistent with the generation of larger numbers of smaller neurons (and thus the observed increase in neuronal density) in the raccoon cerebral cortex, and possibly in the raccoon brain as a whole (**Figure 4**).

### Scaling across Structures

**FIGURE 7:**
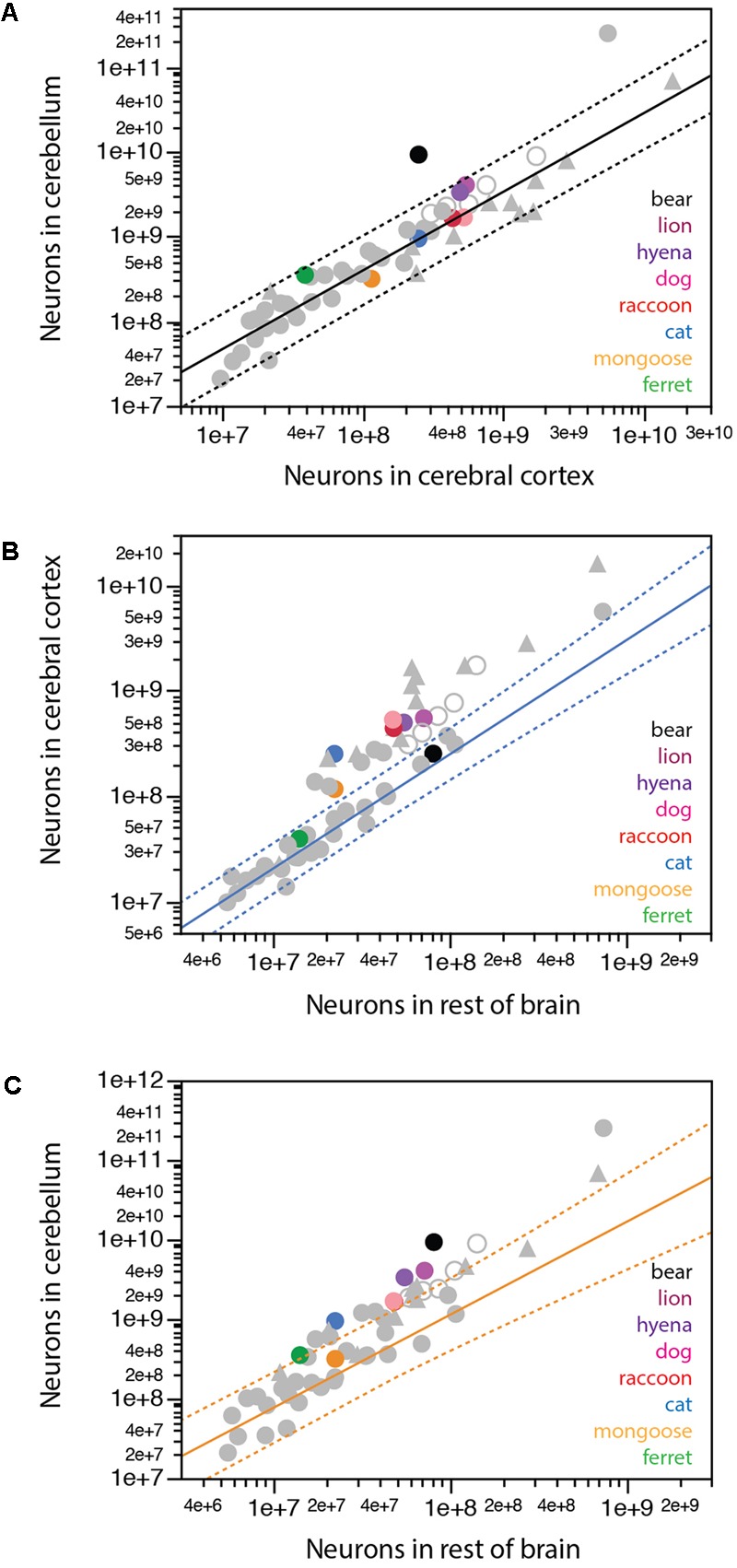
Scaling of numbers of neurons across brain structures in carnivorans. **(A)** Except for the brown bear, carnivorans conform to the relationship between numbers of neurons in the cerebellum and in the cerebral cortex that apply to all mammalian species examined so far, including primates, but excluding the African elephant (exponent, 0.928 ± 0.039, undistinguishable from unity; *r*^2^ = 0.929, *p* < 0.0001, plotted). **(B)** Only the ferret and brown bear conform to the scaling relationship that describes how the number of neurons in the cerebral cortex varies as a power function of the number of neurons in the rest of brain across glires, eulipotyphlans, and small Afrotherians, with exponent 1.085 ± 0.064, undistinguishable from linearity (excluding the African elephant; *r*^2^ = 0.940, *p* < 0.0001, *n* = 20, plotted). All other carnivoran species, like primates, artiodactyls and Australasian marsupials, have more neurons in the cerebral cortex than predicted for the number of neurons in the rest of brain for glires, eulipotyphlans and small Afrotherians. **(C)** Only the banded mongoose among carnivoran species conforms to the scaling relationship that describes how the number of neurons in the cerebellum varies as a power function of the number of neurons in the rest of brain across glires, eulipotyphlans, and small Afrotherians, with exponent 1.170 ± 0.119, undistinguishable from linearity (excluding the African elephant; *r*^2^ = 0.842, *p* < 0.0001, *n* = 20, plotted).

We have previously found that a single power function undistinguishable from linearity and with a slope of around 4.0 describes the relationship between numbers of neurons in the cerebellum and in the cerebral cortex across all mammalian species so far, with the exception of the elephant, which has 44.8 neurons in the cerebellum for every neuron in the cerebral cortex (Herculano-Houzel et al., 2014a). Most carnivorans analyzed conform to the same relationship that applies to other mammals, with the clear exception of the brown bear, which, like the elephant, has a much larger ratio between numbers of neurons in the cerebellum and in the cerebral cortex of 36.9 (**Figure 7A**, black). Remarkably, the lion and hyena also have fewer neurons in the cerebral cortex than expected for their number of neurons in the cerebellum, with 7.4 and 6.7 neurons in the cerebellum for every neuron in the cerebral cortex, in contrast to ratios of 3.8 in the cat and raccoon, 3.2 in the dog and 2.7 in the banded mongoose (**Table 1**).

Whereas the cerebral cortex and cerebellum gain neurons proportionately across the vast majority of mammalian species, a similar concerted scaling on numbers of neurons in the cerebral cortex and rest of brain, with a constant ratio across structures, is true only across glires, eulipotyphlans, small afrotherians, and South American marsupials (Herculano-Houzel, 2016). Across these species, a ratio of 2:1 is maintained between neurons in the cerebral cortex:rest of brain, in what we have proposed to be the ancestral allocation of neurons across these structures (Herculano-Houzel et al., 2014b; Herculano-Houzel, 2016; Dos Santos et al., 2017). Artiodactyls, primates and Australasian marsupials deviate from this relationship, with larger ratios between numbers of neurons in the cerebral cortex and in the rest of brain that may also increase with brain size. We find that among carnivorans, only the ferret and the brown bear align themselves with the first group (**Figure 7B**), with small ratios of 2.7 and 3.1 between numbers of neurons in the cerebral cortex and the rest of brain, respectively, while all other carnivoran species analyzed align with the second group that includes primates and artiodactyls, with larger ratios of 5.1 (mongoose) to 9-11 (raccoon, dog and cat).

In line with the approximately 4:1 ratio between numbers of neurons in the cerebellum and in the cerebral cortex across most species but not the elephant and brown bear, we find that the cerebellum and rest of brain also gain neurons proportionately across glires, eulipotyphlans, small afrotherians, and South American marsupials, maintaining a ratio of about 8:1 (**Figure 7C**). In contrast, artiodactyls, primates and Australasian marsupials gain neurons in the cerebellum faster than in the rest of brain (**Figure 7C**). Carnivorans again align with the latter mammalian species, with faster addition of neurons to the cerebellum than to the rest of brain, and thus larger ratios between numbers of neurons in the two structures, compared to glires, eulipotyphlans, and small afrotherians (**Figure 7C**).

## Discussion

Here we find that all carnivoran species examined match the relationship between brain structure mass and number of non-neuronal cells that has been found to apply to all mammalian species examined so far (Dos Santos et al., 2017; Herculano-Houzel, 2017). This relationship is a consequence of the lack of systematic variation in the density of non-neuronal cells across brain structures and species. Accordingly, none of the eight carnivoran species analyzed deviated significantly from the non-neuronal cell densities found previously in other mammalian species. These findings are consistent with our proposition that the mechanisms that regulate addition of non-neuronal cells to brain tissue have been remarkably conserved in evolution, which indicates that average size of non-neuronal cells is tightly controlled in development and does not accept much variation across species or structures (Mota and Herculano-Houzel, 2014). The addition of non-neuronal cells in development with relatively unchanging cell density across brain structures and species seems to also apply to the raccoon and the brown bear, regardless of the mechanisms that lead to their deviating neuronal densities.

Most of the carnivoran species analyzed also conformed to the relationship between numbers of neurons and neuronal density found to apply to other non-primate species, and thus also to the resulting relationship between numbers of neurons and structure mass (Herculano-Houzel et al., 2014b; Herculano-Houzel, 2017). However, the smallest (ferret) and largest (brown bear) species had fewer neurons in the cerebral cortex than expected for the mass of this structure in a non-primate mammal, a trend followed also by the lion, which has a cortex with fewer neurons than the golden retriever despite being nearly twice larger. As discussed below, in the context of metabolic cost and the relationships across cortical surface area, thickness and number of neurons, the lower than expected neuronal densities restricted to the cerebral cortex are suggestive of neuronal loss. Conversely, the raccoon had systematically larger neuronal densities than expected in all three structures examined – cerebral cortex, cerebellum and rest of brain. In the context of a relationship between cortical surface area and thickness that still matched that found for most other carnivoran species, this finding suggests that the raccoon brain develops with a larger number of smaller neurons than expected for a carnivoran, resulting in larger numbers of neurons than expected for a non-primate, approaching the numbers found in primate species. Indeed, given only the relationship between brain structure mass and numbers of neurons, one might inadvertently take the raccoon for a primate.

### Domestication

Comparisons of the brain mass vs. body mass relationship between domesticated and wild species often yield parallel lines with identical slopes, which have been interpreted as decreased brain size in domesticated animals – that is, a downward shift in the relationship (Kruska, 2007). One should keep in mind, however, that a lateral shift in the relationship is equally possible, with domestication inducing larger body masses rather than decreased brain mass (which would be expected due to greater food availability in captivity). Indeed, recent evidence suggests that domestication of the chicken has led mostly to a larger body mass, and to a lesser extent, to larger (not smaller) absolute brain mass, mainly due to enlargement of the cerebellum (Henriksen et al., 2016). Untangling the two possibilities – increased body mass for the size of the brain or decreased brain mass for the size of the body – is not feasible when brain size and body size are the only variables available, and when only one species is considered in its wild and domesticated versions. By bringing in other variables and examining other carnivoran as well as other non-primate species, here we show that laboratory-raised ferrets as well as the most common domesticated species, cat and dog, do not have smaller brains or fewer neurons than expected for their body mass. Similarly, we have found that the pig shares a relationship between brain mass and number of neurons with other artiodactyl and non-primate species, although it is an outlier in its much larger body mass for its number of brain neurons (Kazu et al., 2014). We thus have no reason to believe that domesticated animals have become any different from other carnivorans in the allometric scaling of their brains (although the ferret, the smallest carnivoran species examined, might be affected by energetic constraints because of its size; see below).

Intraspecific variation is an important issue to consider in this context. We know that it can be large even in species considered to be fairly homogeneous such as the laboratory mouse, in which body mass still varies across young adult animals of same sex and age by a factor of 2, and brain mass varies by a factor of 1.33 (Herculano-Houzel et al., 2015a). Importantly, we found that larger mice do not have significantly larger brains than smaller mice, and those mouse individuals with larger brains or brain structures do not necessarily have more neurons than individuals with smaller brains or brain structures (Herculano-Houzel et al., 2015a). The lack of a strong correlation across individuals mirroring the power functions that apply across species can be attributed to the finding that across mouse individuals, those with more neurons in a brain structure also have *smaller*, not bigger, neurons. This discrepancy suggests a fundamental difference between developmental and evolutionary patterns of brain variation (Herculano-Houzel et al., 2015a). Indeed, it is well established that allometric relationships that apply across species usually do not apply within species, at least not with the same exponents (Armstrong, 1990). The lack of continuity between intra- and interspecific comparisons might still be simply due to the typically much smaller range of variation across individuals of a given species, precluding the calculation of accurate relationships – although that should be possible to compensate for with larger sample sizes. Dogs, with their enormous variation in body and brain size (at least 15-fold and 2-fold, respectively; Wosinski et al., 1996), offer a great opportunity to put to test how brain scaling compares within and across species. While we found that the two dog individuals examined did fit the scaling relationships observed for other carnivoran species, and indeed for many non-primate mammalian species, we couldn’t aim to address the issue of how intraspecific scaling compares to interspecific scaling here due to the difficulty of obtaining large numbers of individuals for a proper study of intraspecific variation.

For all other species, one might be concerned that, except for the raccoon and ferret (*n* = 2 for each), we could only examine a single individual. While we acknowledge that intraspecific variation not only is significant but also is a very interesting topic in its own right, we still expect it to be negligible when compared to the variation over 2 orders of magnitude in body mass across carnivoran species that we examine here. Thus, while expanding the analysis to a larger number of individuals of each species would of course have been ideal, we believe it is reasonable to expect that individual variation in most species other than the dog is unlikely to affect the results we report here.

### Cognitive Implications

We and others have suggested that the absolute number of neurons in the cerebral cortex are a major determinant of the cognitive capabilities of different species (Roth and Dicke, 2005; Herculano-Houzel et al., 2014a; Herculano-Houzel, 2017). Testing that prediction requires data on cognitive performance that can be compared across species. It is only recently that data obtained with systematic comparative analyses have started to become available, although most studies continue to focus on particular clades, mostly primates (Deaner et al., 2007; MacLean et al., 2014; Kabadayi et al., 2016) and birds (MacLean et al., 2014; Kabadayi et al., 2016). Cognitive performance in carnivorans was recently addressed specifically by Benson-Amram et al. (2015). Across these species, even though brain size relative to body mass is a significant predictor of success in opening a puzzle box, species with larger absolute brain volumes also tended to be better than others at opening the puzzle box (Benson-Amram et al., 2015). Other studies found that absolute brain size (or absolute size of the cerebral cortex) across primates is a much better correlate of task performance than encephalization quotient (Deaner et al., 2007; MacLean et al., 2014). Given that larger primate brains are composed of increasing numbers of neurons (Herculano-Houzel et al., 2007; Gabi et al., 2010), improved performance thus correlates with increased numbers of neurons across species, and possibly across clades as well. Indeed, small-brained corvids show similar performance to much larger-brained primates (Kabadayi et al., 2016), which can be explained by their similarly large numbers of pallial neurons despite the difference in brain size (Herculano-Houzel, 2017). It is thus likely that the larger the number of neurons found in the cerebral cortex of a carnivoran, the more cognitively capable the species is.

The twice larger absolute number of neurons we find in the cerebral cortex of the dog compared to the domestic cat suggests that dogs have a cognitive advantage over cats – and raccoons, despite their smaller brain size compared to dogs, should have similar capabilities to dogs. Unfortunately, no dogs, cats or raccoons were examined in the comprehensive study of carnivorans by Benson-Amram et al. (2015), nor were cats and raccoons included amongst the few carnivorans studied by MacLean et al. (2014), only dogs. While our finding of larger numbers of cortical neurons in dogs than in cats may confirm anecdotal perceptions of dog owners and animal trainers as well as unpublished reports that dogs are easier to train and therefore “more intelligent” (Greene, 2011), cat owners would probably protest, and rightly so. Any argument about their cognitive capabilities at this point will be largely a matter of opinion until direct, systematic comparisons of cognitive capacity are performed across these and other species. Moreover, given that both cats and dogs seem to obey the same neuronal scaling rules for the cerebral cortex, any difference in cognitive capabilities between them due to differences in numbers of cortical neurons would be tied to differences in resulting brain size, suggesting that cat-sized dogs, if they have cat-sized brains, might have only as many cortical neurons as domestic cats. Still, our data allow us to predict that, with their larger numbers of neurons in the cerebral cortex, dogs of the sizes examined here should have more complex and flexible cognition than cats.

It was once believed that dogs had evolved special forms of cognition relative to their wild counterparts, wolves (Frank, 1980), but the same author later concluded that his thesis was incorrect and no such difference existed (Frank, 2011). That proposition was, however, taken up by other authors, who argued that dogs had evolved forms of “human-like social cognition” (Hare et al., 2002). However, Wynne (2016) argues that “dogs are better viewed as equipped with the same cognitive skills as many other species, but, living in proximity to and often being totally dependent on human beings, they acquire exquisite sensitivity to human action” (Wynne, 2016). Although the domestic cat has been a favored non-primate model for neurophysiological studies of sensory systems and perception, not much has been done to examine its cognitive capabilities, especially in direct comparison to the domestic dog (Shreve and Udell, 2015). These authors have called attention to how popular articles often present cat cognition with a negative spin, whereas research suggests that domestic cats, like dogs, have developed a range of behaviors that facilitate their interaction with humans. This is an issue that can only be solved by direct comparisons of cognitive capabilities between cats and dogs – though this is only a particular example of how badly needed are systematic comparisons of cognition and behavior across species that can be related to quantitative neuroanatomy (Herculano-Houzel, 2017).

Raccoons have long been considered highly intelligent animals, “shrewd,” “curious” and “mischievous,” and were initially classified as related to the fox, then as species of monkey, until being granted their current status as carnivorans (reviewed in Pettit, 2010). Because of their cognitive “fame,” raccoons became the focus of several studies on their behavior in the early days of psychological research in the beginning of the 20th century (Pettit, 2010). Placing raccoons on a comparative scale with other animals, however, requires direct comparisons of cognitive performance across species that are still lacking. It is interesting to combine our finding that the raccoon is an outlier in its numbers of neurons in all brain structures compared to other non-primates, with larger, primate-like numbers of neurons instead, with the estimate that the raccoon actually has a relatively small prefrontal cortex in comparison to carnivorans with similar or even smaller brain and body sizes (Arsznov and Sakai, 2013). For example, the prefrontal cortex represents only 10% of the raccoon brain volume vs. 20% in the coatimundi, even though the raccoon brain is twice as large as the brain of the coatimundi (Arsznov and Sakai, 2013). It is possible that the large number of cortical neurons and the larger than expected neuronal density in the raccoon are at least partly related to an expanded somatosensory cortex (Welker and Seidenstein, 1959; Welker and Campos, 1963). We are currently examining how numbers of neurons in the prefrontal region of the cortex compare across raccoons and other species, but our current results suggest that the relatively small size of the raccoon prefrontal cortex may be compensated by its unexpectedly high neuronal density, thus resulting in a large absolute number of prefrontal neurons, regardless of an expanded somatosensory cortex.

Along the same lines, we were initially surprised to find that carnivorans align with artiodactyls in the neuronal scaling relationship that applies to their cerebral cortex, such that the lion, a predatorial carnivoran, has only about as many neurons in the cerebral cortex as large artiodactyl species that this species preys upon. The similarity fails to support the existence of positive selective pressure for larger numbers of neurons in predators compared to their prey species, which would presumably be associated with the cognitive requirements of hunting. In this context, however, two possibilities remain that we are now investigating: (1) that similar numbers of neurons are distributed differently in surface area and thickness in carnivorans and artiodactyls, such that the number of functional cortical areas, and therefore cortical cognitive output, may be strikingly different across them; and (2) that for similar numbers of cortical neurons, carnivorans have a larger proportion of these neurons, and therefore a larger absolute number of them, in prefrontal, associative regions involved in goal setting and planning.

### The Largest Carnivoran Cortices Do Not Have the Most Neurons: Evidence of Trade-Off with Body Mass

While being a large carnivoran brings the advantage of not being preyed upon, it comes with a high energetic cost that has been calculated to impose evolutionary constraints on body size in these predators (Carbone et al., 1999, 2007). The large terrestrial carnivorans are constrained to preying on large species, which have limiting low population densities (Carbone et al., 1999), and are further limited by prey biomass and productivity (i.e., prey biomass produced per year; Carbone and Gittleman, 2002). As a result, the largest carnivorans are particularly vulnerable to decreases in prey abundance: these lead to a five to six fold greater decrease in population density of the largest carnivorans compared to the effect on the population density of smaller carnivoran species (Carbone et al., 2011). Similarly, carnivorans must hunt for longer in areas of low prey density or productivity (Carbone et al., 2011).

Hunting in itself is metabolically costly, particularly when hunting for large prey, which requires high speed chases, as the energetic cost of hunting is proportional to chase speed; as a result, large-prey specialists expend about twice as much energy during hunting as a small-prey specialist of same body mass would (Carbone et al., 2007). Hunting large prey is so costly that there is a limit to the additional hunting effort that large carnivorans can still afford to make up for eventual shortage or loss of prey to competitors. For example, losing just 25% of their prey to scavenging hyenas would cause African wild dogs, an average-sized species (ca. 25 kg body mass), to need to increase their daily hunting time from 3.5 h to over 12 h (Gorman et al., 1998). Such an increase which would be physiologically untenable: because of the high metabolic cost of high-speed hunting, African wild dogs already require more than five times the predicted basal daily energy expenditure for a mammal of their body mass, which is close to the calculated physiological limit on sustainable metabolic rates of around 6–7 times the basal metabolic rate (Gorman et al., 1998). Because any increase in the time spent hunting greatly adds to overall energy expenditure, which offsets the possible gains of additional hunting hours, large predatory carnivoran species, with already extremely high hunting costs, are particularly susceptible to changes in feeding ecology. Additionally, the cost of locomotion for the very largest carnivoran species, the lion and polar bear, are 2–3 times higher than expected for mammals of a similar size (Chassin et al., 1976; Hurst et al., 1982).

The brown bear, the largest carnivoran species we analyzed, is omnivorous: this species both eats grass, berries, bulbs and tuber, and hunts. Adding vegetables to their diet, however, is still not enough to make brown bears invulnerable to food availability, as their body mass depends on it. For example, the largest North American brown bears occur in populations that feed on abundant spawning salmon, which does not occur in Europe, and European brown bears are larger in the North than in the South, which appears to be related to greater availability and use of protein-rich meat and insects in the North (Swenson et al., 2007). Female body mass is highly positively correlated with reproductive success across populations, which also indicates that obtaining enough calories is of great consequence (Hildebrand et al., 1999).

Taken together, the energetic costs of being a large carnivoran suggest that balancing their energy budgets requires adjustments that reduce energy expenditure (Carbone et al., 2007). One such adjustment is behavioral inactivity (in which animals may or may not be asleep): lions, for example, spend over 90% of the day inactive (Schaller, 1972). Another feature that minimizes cost is hibernation, which is found in bears and lowers metabolism enough that the body is not damaged by the prolonged anorexia, to the point where not even the expected loss in bone density and muscular mass and strength from prolonged inactivity occur in hibernating bears (Hershey et al., 2008; McGee-Lawrence et al., 2009, 2015).

We suggest that the reduction in the number of neurons in the cerebral cortex of the largest species we examined, the brown bear, and possibly in the lion as well, is related to the large metabolic costs of maintaining a large body mass, and where applicable, needing to spend energy hunting to maintain that mass. We have previously shown that the metabolic cost of the brain is proportional to its number of neurons, regardless of brain size, and that neurons in the cerebral cortex cost on average 10 times as much energy as neurons in the cerebellum (Herculano-Houzel, 2011). Thus, cerebral cortical neurons are expected to be both more vulnerable to caloric shortage than other brain neurons, and to also contribute more to decreasing total metabolic cost when their numbers are reduced than the loss of other neuronal populations would. In this regard, we interpret the finding of a much larger than expected ratio between numbers of neurons in the cerebellum and in the cerebral cortex in the brown bear as a result of an abnormally decreased number of neurons in its cerebral cortex, given that the brown bear has only slightly fewer neurons in the cerebellum than expected for its mass, but far fewer neurons in the cerebral cortex than expected for its mass. This is in contrast to the elephant, the only other exception so far to the average 4 neurons in the cerebellum to every neuron in the cerebral cortex, in which the cerebral cortex fits the expected relationship between number of neurons and structure mass for afrotherians, while the cerebellum has more neurons than expected for its mass (Herculano-Houzel et al., 2014a). The discrepancy in the cerebellum alone indicates that the elephant has an enlarged number of cerebellar neurons, possibly related to somatosensory and motor processing of the trunk (Herculano-Houzel et al., 2014a). A reduced number of cortical neurons in the largest carnivoran species might either be a direct developmental response to caloric shortage, or an evolutionarily incorporated strategy of elimination of cortical neurons that occurs in the largest animals, regardless of their individual developmental history. At the moment we cannot distinguish between these possibilities, although they are not mutually exclusive.

It is possible that the smaller than expected number of cortical neurons found in the brown bear is directly due to hibernation, rather than to the metabolic limitation that leads to hibernation. Differentiating between these two possibilities would require determining cellular composition of the brain in pre- and post-hibernation animals, and in juvenile bears that have never hibernated. However, as mentioned above, the very advantage of hibernation seems to be that metabolism is lowered enough that the body is not damaged by the prolonged anorexia, and thus we find that neuronal loss during this period of lowered metabolic rate would be unlikely, just as there is no loss in bone density of muscular mass in hibernating bears (Hershey et al., 2008; McGee-Lawrence et al., 2009, 2015). Moreover, despite the presumptive thinning of the cortical parenchyma, there were no signs of adult (such as age-related) cortical atrophy, such as gaps between the gyri in the brown bear cortex. Similarly, we found no signs of neuronal loss in another hibernating animal we analyzed previously, the gray squirrel, compared to non-hibernating rodent species (Herculano-Houzel et al., 2011).

Carbone et al. (2007) have suggested that the daily energetic expenditure scales differently with body mass between small and large carnivoran species. Interestingly, the limit between the two groups is at a body mass of 14.5–21 kg, where domestic dogs are found. Domestic dogs have adapted to the starch-rich diet that is typical of modern humans (Axelsson et al., 2013), which might protect them from metabolic constraints that apply to other carnivorans. Importantly, we find that dogs do not have more cortical neurons than predicted for a non-primate of its cortical mass, but just as many as expected. It will be interesting to determine if wolves, with a larger brain size than most domestic dogs, also have as many cortical neurons as predicted, or if there already is evidence that, because of the dependence on hunting, they are subject to a trade-off between body mass and number of cortical neurons as the brown bear and, possibly, the lion.

Remarkably, at the low end of the body mass range of carnivorans, small predators such as the ferret are also expected to face metabolic constraints, due to the costly strategy of feeding on much smaller prey (Carbone et al., 2007). Their higher than expected daily energy expenditure might thus impose a trade-off on the number of cortical neurons found in the ferret cerebral cortex, which we have also found to be lower than expected for the cortical mass of this species, with significantly reduced neuronal densities. Expanding this study to a larger number of carnivoran species spanning the full range of body masses in the clade will help elucidate whether trade-offs between numbers of cortical neurons and body mass do occur as a rule in both the upper and lower limits of body size in carnivorans.

## Author Contributions

SH-H, DJ-M, and PM designed the research; KL, SN, MB, FP, MECL, OM, AA, and PM provided tissue; DJ-M and SH-H collected and analyzed data and wrote the manuscript.

## Conflict of Interest Statement

The authors declare that the research was conducted in the absence of any commercial or financial relationships that could be construed as a potential conflict of interest.
